# (*Z*)-*N*-(1-Eth­oxy­ethyl­idene)-2,6-bis­(propan-2-yl)anilinium chloride hemihydrate

**DOI:** 10.1107/S1600536812037543

**Published:** 2012-09-08

**Authors:** Leila Mokhtabad Amrei, René T. Boeré

**Affiliations:** a Department of Chemistry and Biochemistry, University of Lethbridge, Lethbridge, AB, Canada T1K 3M4

## Abstract

In the title compound, C_16_H_26_NO^+^·Cl^−^·0.5H_2_O, the asymmetric unit consists of two independent cations, their respective chloride anions and a solvent water mol­ecule. Together they form a discrete crescent-shaped entity linked by hydrogen bonds from the central water atom to two Cl^−^ ions and from the latter to two protonated imine groups. The geometries of the two independent cations are essentially the same. The planar N=C(O)CH_3_ groups in each (r.m.s. deviations = 0.0011 and 0.0026 Å) form dihedral angles of 75.28 (5) and 79.10 (4)° with the benzene rings. In one cation, the methyl atoms of one of the isopropyl groups were modeled as disordered over two sets of sites, with refined occupancies of 0.589 (17) and 0.411 (17).

## Related literature

For related structures, see: Shine *et al.* (2004 ▶); Jazzar *et al.* (2007 ▶); Zhang *et al.* (2003 ▶). For hydrogen-bond details, see: Fuller (1959 ▶). For standard geometric data, see: Allen *et al.* (1987 ▶); Orpen & Brammer (1989 ▶). For a description of the Cambridge Structural Database, see: Allen (2002 ▶). For details of the synthesis, see: Boeré *et al.* (1998 ▶).
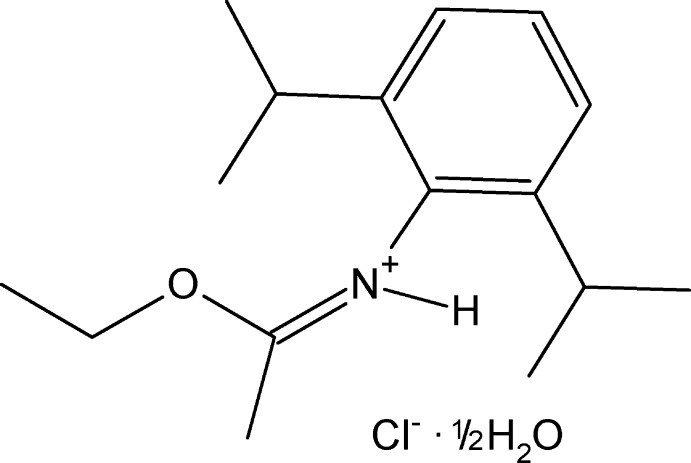


## Experimental

### 

#### Crystal data

C_16_H_26_NO^+^·Cl^−^·0.5H_2_O
*M* *_r_* = 292.84
Triclinic, 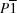

*a* = 11.2193 (7) Å
*b* = 12.9719 (8) Å
*c* = 12.9832 (8) Å
α = 82.637 (1)°
β = 69.831 (1)°
γ = 82.976 (1)°
*V* = 1752.83 (19) Å^3^
*Z* = 4
Mo *K*α radiation
μ = 0.22 mm^−1^
*T* = 173 K
0.45 × 0.20 × 0.10 mm

#### Data collection

Bruker APEXII CCD area-detector diffractometer
Absorption correction: multi-scan (*SADABS*; Bruker, 2008 ▶) *T*_min_ = 0.719, *T*_max_ = 0.746
25695 measured reflections
8002 independent reflections
6041 reflections with *I* > 2σ(*I*)
*R*_int_ = 0.025

#### Refinement

*R*[*F* ^2^ > 2σ(*F* ^2^)] = 0.039
*wR*(*F* ^2^) = 0.101
*S* = 1.04
8002 reflections
395 parameters
H atoms treated by a mixture of independent and constrained refinement
Δρ_max_ = 0.26 e Å^−3^
Δρ_min_ = −0.37 e Å^−3^

### 

Data collection: *APEX2* (Bruker, 2008 ▶); cell refinement: *SAINT-Plus* (Bruker, 2008 ▶); data reduction: *SAINT-Plus*; program(s) used to solve structure: *SHELXS97* (Sheldrick, 2008 ▶); program(s) used to refine structure: *SHELXTL* (Sheldrick, 2008 ▶); molecular graphics: *Mercury* (Macrae *et al.*, 2008 ▶); software used to prepare material for publication: *publCIF* (Westrip, 2010 ▶).

## Supplementary Material

Crystal structure: contains datablock(s) I, global. DOI: 10.1107/S1600536812037543/lh5519sup1.cif

Structure factors: contains datablock(s) I. DOI: 10.1107/S1600536812037543/lh5519Isup2.hkl

Supplementary material file. DOI: 10.1107/S1600536812037543/lh5519Isup3.cml

Additional supplementary materials:  crystallographic information; 3D view; checkCIF report

## Figures and Tables

**Table 1:** Hydrogen-bond geometry (Å, °)

*D*—H⋯*A*	*D*—H	H⋯*A*	*D*⋯*A*	*D*—H⋯*A*
N1—H1*N*⋯Cl1	0.899 (18)	2.178 (19)	3.0737 (13)	173.7 (16)
O1*S*—H2*S*⋯Cl1	0.90 (3)	2.39 (3)	3.2684 (18)	167 (2)
O1*S*—H1*S*⋯Cl2	0.84 (3)	2.37 (3)	3.2026 (18)	176 (2)
N2—H2*N*⋯Cl2	0.94 (2)	2.06 (2)	3.0042 (13)	179.1 (17)

**Fig. 1.**
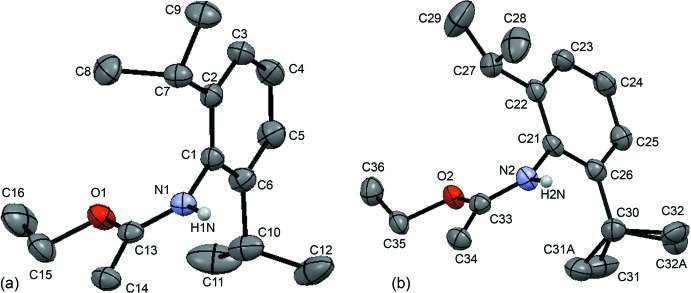
The two independent cations of the title compound with ellipsoids drawn at the 50% probability level. H atoms bonded to C atoms are not shown. Atoms labeled C31/C32/C31A/C32A are the components of disorder.

**Fig. 2.**
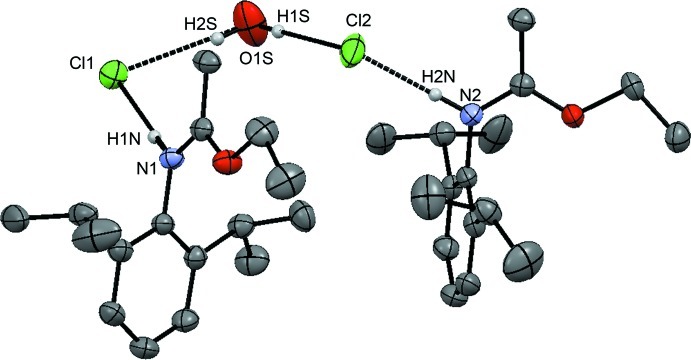
The asymmetric unit of the title compound showing hydrogen bonds as dashed lines. Displacement ellipsoids are drawn at 30% probability level. Neither the disorder nor H atoms bonded to C atoms are shown.

## supplementary crystallographic information

### Comment

The structure of the two independent cations in (I) is shown in Fig. 1. In one cation (*b*) the isopropyl group was refined with a two component disorder model (C31,C32, C31A & C32A). In the structure of 5-(2-((1-ethoxyethylidene)ammonio)-2-methylpropyl)thianthrenium diperchlorate (II) (Shine *et al.*, 2004), which is the only other compound with an ethoxyiminium group that we could locate in the Cambridge Structural Database (Allen, 2002; WebCSD June 2012) (refcode: FARCAD), the geometry about the iminium bond is also *Z*.

The H-bonded cluster, shown in Fig. 2, is the asymmetric unit in the crystal. Both imino nitrogen atoms N1 and N2 are protonated and form strong hydrogen bonds (Table 1) with their respective chloride anions Cl1 and Cl2, within the s.u. of the expected N···Cl distance of 3.19 (7) Å (Fuller, 1959) but clearly on the short end of this scale. The N1···Cl1 bond is comparable in strength to that between N1 and Cl1 in the crystal structure of 2,6-di-isopropyl-*N*-((1-(2-methylprop-2-en-1-yl)cyclohexyl)methylidene)anilinium chloride chloroform solvate (III) (Refcode: GIBLAF) at 3.077 (4) Å (Jazzar *et al.*, 2007); and shorter than N1···Br1 in 2-((2,6-diisopropylphenyl)iminio)propyl bromide (IV), (Refcode: OJIKX) at 3.196 (1) Å (Zhang *et al.*, 2003). There are additional hydrogen bonds in structure (I) between the the two water hydrogen atoms and the chloride anions (Table 1 & Figure 2).

The iminium bond lengths N1═C13, 1.2956 (18) Å and N2═C33, 1.2945 (18) Å, fit well with standard values for C═N of 1.287 (21) Å, whereas the standard value for C—N is 1.479 (36) Å, (Orpen *et al.*, 1989). Comparative N═C values occur in structures (II), 1.289 (5), (III), 1.289 (4) and (IV), 1.290 (2) Å. In (I), the O1—C13 bond length is 1.3068 (17) Å and O2—C33 is 1.3062 (17) Å, indicative of partial double bonds (standard bond lengths for C—O are 1.43 (1) Å, for C═O, 1.23 (1) Å, and for C*sp*^2^—O, 1.354 (16) Å (Allen *et al.*, 1987)).

### Experimental

The title compound (I) was obtained as a side product of the reaction of (2,6-diisopropylphenyl)phosphine with *N*-[2,6-bis(1-methylethyl)phenyl]-2,2-dimethylmethanimidoyl chloride (Boeré *et al.*, 1998) in THF solution (24 h reflux). While the main product precipitated after cooling, the filtrate was evaporated and treated with ethanol. On cooling this mixture to 243 K for an extended period, crystals of (I) formed as colourless blocks. Presumably, (I) forms by the direct nucleophilic addition of ethanol to unreacted imidoyl chloride. It crystallizes as a hemi-hydrate.

### Refinement

During refinement, the methyl groups of one isopropyl group on each of the cations, specifically C8 & C9 and C31 & C32, evidenced large anisotropic displacements. Attempts to define a two-position disorder model for C8 & C9 failed, so these atoms have been treated by a straightforward anisotropic refinement. However, a very reasonable chemical model ensued from a two-position disorder model for C31 and C32 (refined occupancy ratio of 0.589 (17):0.411 (17)). Attempts to include C30 within the disorder model did not lead to a stable refinement, so the second methine carbon was geometrically defined by a dummy atom to ensure the generation of the disordered H atoms on this atom. Hydrogen atoms attached to carbon were treated as riding, with C—H = 0.98 Å and *U*_iso_(H) = 1.5*U*_eq_(C) for methyl, C—H = 1.00 Å and *U*_iso_(H) = 1.2*U*_eq_(C) for methine and C—H = 0.95 Å and *U*_iso_(H) = 1.2*U*_eq_(C) for aromatic H atoms. The NH and OH hydrogen positions are freely refined with isotropic displacements.

### Crystal data

**Table:**

C_16_H_26_NO^+^·Cl^−^·0.5H_2_O	*Z* = 4
*M**_r_* = 292.84	*F*(000) = 636
Triclinic, *P*1	*D*_x_ = 1.110 Mg m^−^^3^
Hall symbol: -P 1	Mo *K*α radiation, λ = 0.71073 Å
*a* = 11.2193 (7) Å	Cell parameters from 9981 reflections
*b* = 12.9719 (8) Å	θ = 2.4–27.5°
*c* = 12.9832 (8) Å	µ = 0.22 mm^−^^1^
α = 82.637 (1)°	*T* = 173 K
β = 69.831 (1)°	Block, colourless
γ = 82.976 (1)°	0.45 × 0.20 × 0.10 mm
*V* = 1752.83 (19) Å^3^	

### Data collection

**Table:**

Bruker APEXII CCD area-detector diffractometer	8002 independent reflections
Radiation source: fine-focus sealed tube, Bruker D8	6041 reflections with *I* > 2σ(*I*)
Graphite monochromator	*R*_int_ = 0.025
Detector resolution: 66.06 pixels mm^-1^	θ_max_ = 27.5°, θ_min_ = 1.6°
φ and ω scans	*h* = −14→14
Absorption correction: multi-scan (*SADABS*; Bruker, 2008)	*k* = −16→16
*T*_min_ = 0.719, *T*_max_ = 0.746	*l* = −16→16
25695 measured reflections	

### Refinement

**Table:**

Refinement on *F*^2^	Primary atom site location: structure-invariant direct methods
Least-squares matrix: full	Secondary atom site location: difference Fourier map
*R*[*F*^2^ > 2σ(*F*^2^)] = 0.039	Hydrogen site location: inferred from neighbouring sites
*wR*(*F*^2^) = 0.101	H atoms treated by a mixture of independent and constrained refinement
*S* = 1.04	*w* = 1/[σ^2^(*F*_o_^2^) + (0.0404*P*)^2^ + 0.4642*P*] where *P* = (*F*_o_^2^ + 2*F*_c_^2^)/3
8002 reflections	(Δ/σ)_max_ = 0.001
395 parameters	Δρ_max_ = 0.26 e Å^−^^3^
0 restraints	Δρ_min_ = −0.37 e Å^−^^3^

### Special details

**Table:**

Experimental. A crystal coated in Paratone (TM) oil was mounted on the end of a thin glass capillary and cooled in the gas stream of the diffractometer Kryoflex device.
Geometry. All e.s.d.'s (except the e.s.d. in the dihedral angle between two l.s. planes) are estimated using the full covariance matrix. The cell e.s.d.'s are taken into account individually in the estimation of e.s.d.'s in distances, angles and torsion angles; correlations between e.s.d.'s in cell parameters are only used when they are defined by crystal symmetry. An approximate (isotropic) treatment of cell e.s.d.'s is used for estimating e.s.d.'s involving l.s. planes.
Refinement. Refinement of *F*^2^ against ALL reflections. The weighted *R*-factor *wR* and goodness of fit *S* are based on *F*^2^, conventional *R*-factors *R* are based on *F*, with *F* set to zero for negative *F*^2^. The threshold expression of *F*^2^ > σ(*F*^2^) is used only for calculating *R*-factors(gt) *etc*. and is not relevant to the choice of reflections for refinement. *R*-factors based on *F*^2^ are statistically about twice as large as those based on *F*, and *R*– factors based on ALL data will be even larger.

### Fractional atomic coordinates and isotropic or equivalent isotropic displacement parameters (Å^2^)

**Table:**

	*x*	*y*	*z*	*U*_iso_*/*U*_eq_	Occ. (<1)
Cl1	−0.09534 (4)	0.79658 (3)	0.07415 (3)	0.03838 (10)	
O1	0.18322 (10)	1.02081 (8)	0.16965 (8)	0.0346 (2)	
N1	0.02336 (11)	0.92678 (9)	0.18958 (9)	0.0265 (2)	
H1N	−0.0157 (17)	0.8872 (14)	0.1610 (14)	0.045 (5)*	
C1	−0.03941 (13)	0.94616 (11)	0.30409 (11)	0.0269 (3)	
C2	−0.10664 (14)	1.04286 (12)	0.32881 (12)	0.0318 (3)	
C3	−0.16881 (15)	1.05703 (13)	0.43971 (12)	0.0378 (4)	
H3A	−0.2155	1.1218	0.4597	0.045*	
C4	−0.16388 (15)	0.97895 (13)	0.52075 (12)	0.0396 (4)	
H4A	−0.2058	0.9908	0.5960	0.048*	
C5	−0.09862 (14)	0.88350 (13)	0.49387 (12)	0.0353 (3)	
H5A	−0.0971	0.8301	0.5509	0.042*	
C6	−0.03497 (13)	0.86427 (11)	0.38452 (11)	0.0279 (3)	
C7	−0.11654 (17)	1.13018 (12)	0.24190 (13)	0.0427 (4)	
H7A	−0.0659	1.1055	0.1682	0.051*	
C8	−0.0590 (3)	1.22705 (15)	0.25371 (18)	0.0764 (7)	
H8A	0.0295	1.2086	0.2505	0.115*	
H8B	−0.1083	1.2542	0.3245	0.115*	
H8C	−0.0612	1.2805	0.1936	0.115*	
C9	−0.2546 (2)	1.15397 (19)	0.24565 (16)	0.0708 (7)	
H9A	−0.2878	1.0901	0.2375	0.106*	
H9B	−0.2585	1.2068	0.1855	0.106*	
H9C	−0.3063	1.1803	0.3164	0.106*	
C10	0.03973 (14)	0.76086 (11)	0.35270 (12)	0.0328 (3)	
H10A	0.0297	0.7460	0.2826	0.039*	
C11	0.18162 (16)	0.76757 (13)	0.33031 (15)	0.0443 (4)	
H11A	0.2113	0.8269	0.2761	0.066*	
H11B	0.2294	0.7030	0.3015	0.066*	
H11C	0.1952	0.7771	0.3990	0.066*	
C12	−0.00771 (19)	0.66928 (13)	0.43852 (14)	0.0477 (4)	
H12A	−0.0993	0.6667	0.4545	0.072*	
H12B	0.0082	0.6785	0.5063	0.072*	
H12C	0.0376	0.6040	0.4097	0.072*	
C13	0.12891 (13)	0.96362 (10)	0.12563 (11)	0.0277 (3)	
C14	0.18325 (15)	0.94020 (12)	0.00923 (12)	0.0345 (3)	
H14A	0.1262	0.8983	−0.0072	0.052*	
H14B	0.2668	0.9012	−0.0035	0.052*	
H14C	0.1928	1.0056	−0.0388	0.052*	
C15	0.30280 (16)	1.06551 (14)	0.10098 (14)	0.0464 (4)	
H15A	0.2896	1.1126	0.0388	0.056*	
H15B	0.3695	1.0094	0.0708	0.056*	
C16	0.3416 (2)	1.12490 (16)	0.17405 (16)	0.0605 (5)	
H16A	0.4182	1.1600	0.1304	0.091*	
H16B	0.3597	1.0766	0.2324	0.091*	
H16C	0.2724	1.1770	0.2072	0.091*	
Cl2	0.40771 (4)	0.73504 (4)	0.03663 (4)	0.05588 (14)	
O2	0.74356 (9)	0.55679 (8)	0.19207 (8)	0.0308 (2)	
N2	0.56122 (11)	0.62883 (10)	0.17525 (10)	0.0298 (3)	
H2N	0.5132 (18)	0.6616 (15)	0.1312 (16)	0.056 (5)*	
C21	0.49447 (13)	0.61613 (11)	0.29274 (11)	0.0280 (3)	
C22	0.50687 (13)	0.68840 (12)	0.35873 (12)	0.0310 (3)	
C23	0.43817 (14)	0.67436 (13)	0.47090 (12)	0.0355 (3)	
H23A	0.4441	0.7216	0.5188	0.043*	
C24	0.36165 (14)	0.59286 (13)	0.51361 (12)	0.0371 (4)	
H24A	0.3166	0.5841	0.5905	0.045*	
C25	0.34996 (14)	0.52411 (12)	0.44569 (12)	0.0345 (3)	
H25A	0.2963	0.4688	0.4764	0.041*	
C26	0.41570 (13)	0.53450 (11)	0.33257 (11)	0.0296 (3)	
C27	0.58849 (15)	0.77944 (13)	0.31014 (14)	0.0393 (4)	
H27A	0.6627	0.7550	0.2468	0.047*	
C28	0.5143 (2)	0.86886 (15)	0.2643 (2)	0.0660 (6)	
H28A	0.4825	0.8434	0.2116	0.099*	
H28B	0.5704	0.9244	0.2270	0.099*	
H28C	0.4423	0.8963	0.3248	0.099*	
C29	0.6417 (2)	0.81701 (19)	0.3904 (2)	0.0745 (7)	
H29A	0.6876	0.7582	0.4200	0.112*	
H29B	0.5716	0.8465	0.4510	0.112*	
H29C	0.7002	0.8706	0.3520	0.112*	
C33	0.67989 (13)	0.59858 (11)	0.12759 (11)	0.0280 (3)	
C34	0.73662 (15)	0.61067 (12)	0.00587 (11)	0.0343 (3)	
H34A	0.6702	0.6375	−0.0260	0.051*	
H34B	0.7744	0.5428	−0.0209	0.051*	
H34C	0.8028	0.6597	−0.0160	0.051*	
C35	0.87984 (13)	0.52399 (12)	0.14489 (12)	0.0333 (3)	
H35A	0.9228	0.5754	0.0842	0.040*	
H35B	0.8920	0.4552	0.1156	0.040*	
C36	0.93303 (16)	0.51755 (14)	0.23654 (14)	0.0438 (4)	
H36A	1.0246	0.4967	0.2089	0.066*	
H36B	0.8902	0.4659	0.2956	0.066*	
H36C	0.9191	0.5859	0.2653	0.066*	
C30	0.40387 (15)	0.45877 (12)	0.25679 (12)	0.0352 (3)	
H30A	0.4524	0.4832	0.1789	0.042*	0.411 (17)
C31	0.4631 (17)	0.3464 (9)	0.2857 (13)	0.070 (3)	0.411 (17)
H31A	0.4651	0.3000	0.2311	0.106*	0.411 (17)
H31B	0.4109	0.3186	0.3592	0.106*	0.411 (17)
H31C	0.5500	0.3509	0.2849	0.106*	0.411 (17)
C32	0.2661 (14)	0.4547 (10)	0.2676 (11)	0.048 (2)	0.411 (17)
H32A	0.2342	0.5218	0.2386	0.073*	0.411 (17)
H32B	0.2154	0.4403	0.3454	0.073*	0.411 (17)
H32C	0.2593	0.3992	0.2257	0.073*	0.411 (17)
H30B	0.4196	0.4980	0.1824	0.042*	0.589 (17)
C31A	0.5042 (6)	0.3704 (6)	0.2436 (6)	0.0495 (13)	0.589 (17)
H31D	0.5882	0.3976	0.2187	0.074*	0.589 (17)
H31E	0.4996	0.3267	0.1890	0.074*	0.589 (17)
H31F	0.4908	0.3285	0.3145	0.074*	0.589 (17)
C32A	0.2725 (9)	0.4191 (8)	0.2905 (9)	0.0563 (19)	0.589 (17)
H32D	0.2663	0.3838	0.2304	0.085*	0.589 (17)
H32E	0.2073	0.4780	0.3062	0.085*	0.589 (17)
H32F	0.2592	0.3699	0.3566	0.085*	0.589 (17)
O1S	0.16647 (17)	0.64100 (12)	0.01655 (15)	0.0698 (5)	
H1S	0.230 (2)	0.6630 (19)	0.0241 (19)	0.079 (8)*	
H2S	0.102 (3)	0.691 (2)	0.034 (2)	0.100 (9)*	

### Atomic displacement parameters (Å^2^)

**Table:**

	*U*^11^	*U*^22^	*U*^33^	*U*^12^	*U*^13^	*U*^23^
Cl1	0.0419 (2)	0.0331 (2)	0.0441 (2)	−0.00507 (16)	−0.01803 (17)	−0.00555 (16)
O1	0.0375 (6)	0.0351 (6)	0.0301 (5)	−0.0114 (5)	−0.0076 (4)	−0.0014 (4)
N1	0.0308 (6)	0.0248 (6)	0.0243 (6)	−0.0013 (5)	−0.0092 (5)	−0.0049 (5)
C1	0.0260 (7)	0.0314 (8)	0.0234 (7)	−0.0030 (6)	−0.0072 (5)	−0.0050 (6)
C2	0.0349 (8)	0.0327 (8)	0.0286 (7)	0.0021 (6)	−0.0121 (6)	−0.0067 (6)
C3	0.0396 (9)	0.0393 (9)	0.0329 (8)	0.0082 (7)	−0.0107 (7)	−0.0122 (7)
C4	0.0389 (9)	0.0507 (10)	0.0248 (7)	0.0021 (7)	−0.0051 (6)	−0.0090 (7)
C5	0.0355 (8)	0.0424 (9)	0.0259 (7)	−0.0043 (7)	−0.0090 (6)	0.0016 (6)
C6	0.0258 (7)	0.0308 (8)	0.0283 (7)	−0.0040 (6)	−0.0099 (6)	−0.0027 (6)
C7	0.0603 (11)	0.0339 (9)	0.0311 (8)	0.0143 (8)	−0.0161 (8)	−0.0078 (7)
C8	0.145 (2)	0.0294 (10)	0.0560 (12)	−0.0041 (12)	−0.0377 (14)	−0.0001 (9)
C9	0.0715 (14)	0.0921 (17)	0.0383 (10)	0.0476 (12)	−0.0203 (10)	−0.0139 (10)
C10	0.0395 (8)	0.0288 (8)	0.0297 (7)	−0.0011 (6)	−0.0120 (6)	−0.0017 (6)
C11	0.0385 (9)	0.0384 (9)	0.0515 (10)	0.0077 (7)	−0.0130 (8)	−0.0045 (8)
C12	0.0624 (12)	0.0332 (9)	0.0445 (10)	−0.0063 (8)	−0.0162 (9)	0.0044 (7)
C13	0.0331 (7)	0.0222 (7)	0.0263 (7)	0.0026 (6)	−0.0103 (6)	−0.0004 (5)
C14	0.0415 (8)	0.0308 (8)	0.0259 (7)	0.0017 (6)	−0.0062 (6)	−0.0019 (6)
C15	0.0422 (10)	0.0514 (11)	0.0410 (9)	−0.0198 (8)	−0.0068 (7)	0.0075 (8)
C16	0.0661 (13)	0.0613 (13)	0.0587 (12)	−0.0375 (11)	−0.0208 (10)	0.0085 (10)
Cl2	0.0394 (2)	0.0776 (3)	0.0490 (3)	−0.0002 (2)	−0.0218 (2)	0.0156 (2)
O2	0.0247 (5)	0.0352 (6)	0.0291 (5)	0.0025 (4)	−0.0068 (4)	−0.0020 (4)
N2	0.0275 (6)	0.0353 (7)	0.0250 (6)	0.0014 (5)	−0.0088 (5)	−0.0010 (5)
C21	0.0222 (7)	0.0353 (8)	0.0238 (7)	0.0046 (6)	−0.0067 (5)	−0.0030 (6)
C22	0.0226 (7)	0.0352 (8)	0.0339 (8)	0.0021 (6)	−0.0087 (6)	−0.0051 (6)
C23	0.0306 (8)	0.0439 (9)	0.0327 (8)	0.0017 (7)	−0.0098 (6)	−0.0125 (7)
C24	0.0319 (8)	0.0487 (10)	0.0262 (7)	0.0002 (7)	−0.0044 (6)	−0.0056 (7)
C25	0.0308 (8)	0.0375 (8)	0.0319 (8)	−0.0032 (6)	−0.0073 (6)	−0.0005 (6)
C26	0.0267 (7)	0.0329 (8)	0.0287 (7)	0.0038 (6)	−0.0106 (6)	−0.0035 (6)
C27	0.0307 (8)	0.0415 (9)	0.0435 (9)	−0.0058 (7)	−0.0068 (7)	−0.0094 (7)
C28	0.0588 (13)	0.0438 (11)	0.0941 (17)	−0.0141 (9)	−0.0267 (12)	0.0105 (11)
C29	0.0741 (15)	0.0854 (17)	0.0793 (15)	−0.0415 (13)	−0.0361 (13)	−0.0002 (13)
C33	0.0294 (7)	0.0264 (7)	0.0279 (7)	−0.0030 (6)	−0.0091 (6)	−0.0025 (6)
C34	0.0351 (8)	0.0374 (8)	0.0269 (7)	−0.0024 (7)	−0.0060 (6)	−0.0037 (6)
C35	0.0228 (7)	0.0328 (8)	0.0399 (8)	0.0027 (6)	−0.0061 (6)	−0.0046 (7)
C36	0.0331 (8)	0.0515 (10)	0.0490 (10)	−0.0025 (7)	−0.0173 (7)	−0.0039 (8)
C30	0.0369 (8)	0.0381 (9)	0.0315 (8)	−0.0027 (7)	−0.0121 (7)	−0.0048 (6)
C31	0.103 (9)	0.047 (5)	0.090 (7)	0.024 (5)	−0.069 (7)	−0.031 (5)
C32	0.053 (4)	0.051 (6)	0.055 (5)	0.000 (5)	−0.031 (3)	−0.023 (4)
C30A	0.0369 (8)	0.0381 (9)	0.0315 (8)	−0.0027 (7)	−0.0121 (7)	−0.0048 (6)
C31A	0.052 (3)	0.043 (3)	0.056 (3)	0.0078 (19)	−0.020 (2)	−0.020 (2)
C32A	0.039 (2)	0.062 (5)	0.071 (5)	−0.003 (3)	−0.015 (3)	−0.027 (3)
O1S	0.0513 (9)	0.0430 (8)	0.1182 (14)	−0.0026 (7)	−0.0256 (9)	−0.0280 (8)

### Geometric parameters (Å, º)

**Table:**

O1—C13	1.3068 (17)	N2—H2N	0.94 (2)
O1—C15	1.4711 (18)	C21—C26	1.394 (2)
N1—C13	1.2956 (18)	C21—C22	1.397 (2)
N1—C1	1.4490 (17)	C22—C23	1.393 (2)
N1—H1N	0.899 (18)	C22—C27	1.519 (2)
C1—C2	1.397 (2)	C23—C24	1.380 (2)
C1—C6	1.4000 (19)	C23—H23A	0.9500
C2—C3	1.392 (2)	C24—C25	1.378 (2)
C2—C7	1.515 (2)	C24—H24A	0.9500
C3—C4	1.375 (2)	C25—C26	1.395 (2)
C3—H3A	0.9500	C25—H25A	0.9500
C4—C5	1.380 (2)	C26—C30	1.523 (2)
C4—H4A	0.9500	C27—C29	1.520 (3)
C5—C6	1.391 (2)	C27—C28	1.524 (3)
C5—H5A	0.9500	C27—H27A	1.0000
C6—C10	1.517 (2)	C28—H28A	0.9800
C7—C9	1.528 (3)	C28—H28B	0.9800
C7—C8	1.528 (3)	C28—H28C	0.9800
C7—H7A	1.0000	C29—H29A	0.9800
C8—H8A	0.9800	C29—H29B	0.9800
C8—H8B	0.9800	C29—H29C	0.9800
C8—H8C	0.9800	C33—C34	1.4813 (19)
C9—H9A	0.9800	C34—H34A	0.9800
C9—H9B	0.9800	C34—H34B	0.9800
C9—H9C	0.9800	C34—H34C	0.9800
C10—C11	1.528 (2)	C35—C36	1.494 (2)
C10—C12	1.530 (2)	C35—H35A	0.9900
C10—H10A	1.0000	C35—H35B	0.9900
C11—H11A	0.9800	C36—H36A	0.9800
C11—H11B	0.9800	C36—H36B	0.9800
C11—H11C	0.9800	C36—H36C	0.9800
C12—H12A	0.9800	C30—C32	1.510 (14)
C12—H12B	0.9800	C30—C31	1.582 (10)
C12—H12C	0.9800	C30—H30A	1.0000
C13—C14	1.4781 (19)	C31—H31A	0.9800
C14—H14A	0.9800	C31—H31B	0.9800
C14—H14B	0.9800	C31—H31C	0.9800
C14—H14C	0.9800	C32—H32A	0.9800
C15—C16	1.496 (3)	C32—H32B	0.9800
C15—H15A	0.9900	C32—H32C	0.9800
C15—H15B	0.9900	C31A—H31D	0.9800
C16—H16A	0.9800	C31A—H31E	0.9800
C16—H16B	0.9800	C31A—H31F	0.9800
C16—H16C	0.9800	C32A—H32D	0.9800
O2—C33	1.3062 (17)	C32A—H32E	0.9800
O2—C35	1.4682 (16)	C32A—H32F	0.9800
N2—C33	1.2945 (18)	O1S—H1S	0.84 (3)
N2—C21	1.4467 (17)	O1S—H2S	0.90 (3)
			
C13—O1—C15	119.20 (12)	H16A—C16—H16C	109.5
C13—N1—C1	125.12 (12)	H16B—C16—H16C	109.5
C13—N1—H1N	118.2 (11)	C33—O2—C35	120.00 (11)
C1—N1—H1N	116.7 (11)	C33—N2—C21	124.87 (12)
C2—C1—C6	123.22 (13)	C33—N2—H2N	118.7 (11)
C2—C1—N1	118.87 (12)	C21—N2—H2N	116.4 (11)
C6—C1—N1	117.81 (12)	C26—C21—C22	123.76 (13)
C3—C2—C1	116.99 (13)	C26—C21—N2	117.26 (13)
C3—C2—C7	119.52 (14)	C22—C21—N2	118.90 (13)
C1—C2—C7	123.48 (13)	C23—C22—C21	116.73 (14)
C4—C3—C2	121.16 (15)	C23—C22—C27	121.61 (14)
C4—C3—H3A	119.4	C21—C22—C27	121.65 (13)
C2—C3—H3A	119.4	C24—C23—C22	121.05 (14)
C3—C4—C5	120.61 (14)	C24—C23—H23A	119.5
C3—C4—H4A	119.7	C22—C23—H23A	119.5
C5—C4—H4A	119.7	C25—C24—C23	120.62 (14)
C4—C5—C6	121.00 (14)	C25—C24—H24A	119.7
C4—C5—H5A	119.5	C23—C24—H24A	119.7
C6—C5—H5A	119.5	C24—C25—C26	121.00 (14)
C5—C6—C1	116.98 (13)	C24—C25—H25A	119.5
C5—C6—C10	122.12 (13)	C26—C25—H25A	119.5
C1—C6—C10	120.87 (12)	C21—C26—C25	116.80 (13)
C2—C7—C9	110.52 (15)	C21—C26—C30	121.73 (13)
C2—C7—C8	111.11 (14)	C25—C26—C30	121.46 (13)
C9—C7—C8	112.05 (17)	C22—C27—C29	113.57 (15)
C2—C7—H7A	107.7	C22—C27—C28	110.28 (13)
C9—C7—H7A	107.7	C29—C27—C28	110.76 (17)
C8—C7—H7A	107.7	C22—C27—H27A	107.3
C7—C8—H8A	109.5	C29—C27—H27A	107.3
C7—C8—H8B	109.5	C28—C27—H27A	107.3
H8A—C8—H8B	109.5	C27—C28—H28A	109.5
C7—C8—H8C	109.5	C27—C28—H28B	109.5
H8A—C8—H8C	109.5	H28A—C28—H28B	109.5
H8B—C8—H8C	109.5	C27—C28—H28C	109.5
C7—C9—H9A	109.5	H28A—C28—H28C	109.5
C7—C9—H9B	109.5	H28B—C28—H28C	109.5
H9A—C9—H9B	109.5	C27—C29—H29A	109.5
C7—C9—H9C	109.5	C27—C29—H29B	109.5
H9A—C9—H9C	109.5	H29A—C29—H29B	109.5
H9B—C9—H9C	109.5	C27—C29—H29C	109.5
C6—C10—C11	110.72 (12)	H29A—C29—H29C	109.5
C6—C10—C12	113.39 (13)	H29B—C29—H29C	109.5
C11—C10—C12	109.74 (14)	N2—C33—O2	116.56 (12)
C6—C10—H10A	107.6	N2—C33—C34	120.19 (13)
C11—C10—H10A	107.6	O2—C33—C34	123.24 (13)
C12—C10—H10A	107.6	C33—C34—H34A	109.5
C10—C11—H11A	109.5	C33—C34—H34B	109.5
C10—C11—H11B	109.5	H34A—C34—H34B	109.5
H11A—C11—H11B	109.5	C33—C34—H34C	109.5
C10—C11—H11C	109.5	H34A—C34—H34C	109.5
H11A—C11—H11C	109.5	H34B—C34—H34C	109.5
H11B—C11—H11C	109.5	O2—C35—C36	106.30 (12)
C10—C12—H12A	109.5	O2—C35—H35A	110.5
C10—C12—H12B	109.5	C36—C35—H35A	110.5
H12A—C12—H12B	109.5	O2—C35—H35B	110.5
C10—C12—H12C	109.5	C36—C35—H35B	110.5
H12A—C12—H12C	109.5	H35A—C35—H35B	108.7
H12B—C12—H12C	109.5	C35—C36—H36A	109.5
N1—C13—O1	116.65 (12)	C35—C36—H36B	109.5
N1—C13—C14	120.48 (13)	H36A—C36—H36B	109.5
O1—C13—C14	122.86 (13)	C35—C36—H36C	109.5
C13—C14—H14A	109.5	H36A—C36—H36C	109.5
C13—C14—H14B	109.5	H36B—C36—H36C	109.5
H14A—C14—H14B	109.5	C32—C30—C26	110.7 (5)
C13—C14—H14C	109.5	C32—C30—C31	110.1 (5)
H14A—C14—H14C	109.5	C26—C30—C31	109.7 (4)
H14B—C14—H14C	109.5	C32—C30—H30A	108.8
O1—C15—C16	106.63 (14)	C26—C30—H30A	108.8
O1—C15—H15A	110.4	C31—C30—H30A	108.8
C16—C15—H15A	110.4	H31D—C31A—H31E	109.5
O1—C15—H15B	110.4	H31D—C31A—H31F	109.5
C16—C15—H15B	110.4	H31E—C31A—H31F	109.5
H15A—C15—H15B	108.6	H32D—C32A—H32E	109.5
C15—C16—H16A	109.5	H32D—C32A—H32F	109.5
C15—C16—H16B	109.5	H32E—C32A—H32F	109.5
H16A—C16—H16B	109.5	H1S—O1S—H2S	107 (2)
C15—C16—H16C	109.5		
			
C13—N1—C1—C2	76.02 (18)	C33—N2—C21—C26	−101.16 (17)
C13—N1—C1—C6	−107.32 (16)	C33—N2—C21—C22	82.07 (18)
C6—C1—C2—C3	1.6 (2)	C26—C21—C22—C23	1.9 (2)
N1—C1—C2—C3	178.04 (13)	N2—C21—C22—C23	178.40 (12)
C6—C1—C2—C7	−177.25 (14)	C26—C21—C22—C27	−176.59 (14)
N1—C1—C2—C7	−0.8 (2)	N2—C21—C22—C27	−0.1 (2)
C1—C2—C3—C4	−0.1 (2)	C21—C22—C23—C24	−0.2 (2)
C7—C2—C3—C4	178.82 (15)	C27—C22—C23—C24	178.27 (14)
C2—C3—C4—C5	−1.1 (3)	C22—C23—C24—C25	−1.0 (2)
C3—C4—C5—C6	0.8 (2)	C23—C24—C25—C26	0.5 (2)
C4—C5—C6—C1	0.6 (2)	C22—C21—C26—C25	−2.3 (2)
C4—C5—C6—C10	178.73 (14)	N2—C21—C26—C25	−178.85 (12)
C2—C1—C6—C5	−1.9 (2)	C22—C21—C26—C30	178.97 (13)
N1—C1—C6—C5	−178.35 (12)	N2—C21—C26—C30	2.38 (19)
C2—C1—C6—C10	−179.99 (13)	C24—C25—C26—C21	1.0 (2)
N1—C1—C6—C10	3.51 (19)	C24—C25—C26—C30	179.78 (14)
C3—C2—C7—C9	−62.8 (2)	C23—C22—C27—C29	29.2 (2)
C1—C2—C7—C9	115.95 (17)	C21—C22—C27—C29	−152.44 (16)
C3—C2—C7—C8	62.2 (2)	C23—C22—C27—C28	−95.80 (19)
C1—C2—C7—C8	−119.01 (18)	C21—C22—C27—C28	82.57 (19)
C5—C6—C10—C11	−97.83 (16)	C21—N2—C33—O2	−2.4 (2)
C1—C6—C10—C11	80.21 (17)	C21—N2—C33—C34	176.78 (13)
C5—C6—C10—C12	26.0 (2)	C35—O2—C33—N2	−177.72 (12)
C1—C6—C10—C12	−155.93 (14)	C35—O2—C33—C34	3.2 (2)
C1—N1—C13—O1	1.5 (2)	C33—O2—C35—C36	159.67 (13)
C1—N1—C13—C14	−178.89 (13)	C21—C26—C30—C32	−125.3 (5)
C15—O1—C13—N1	−179.54 (13)	C25—C26—C30—C32	56.0 (6)
C15—O1—C13—C14	0.8 (2)	C21—C26—C30—C31	112.9 (8)
C13—O1—C15—C16	−179.29 (14)	C25—C26—C30—C31	−65.8 (8)

### Hydrogen-bond geometry (Å, º)

**Table:**

*D*—H···*A*	*D*—H	H···*A*	*D*···*A*	*D*—H···*A*
N1—H1*N*···Cl1	0.899 (18)	2.178 (19)	3.0737 (13)	173.7 (16)
O1*S*—H2*S*···Cl1	0.90 (3)	2.39 (3)	3.2684 (18)	167 (2)
O1*S*—H1*S*···Cl2	0.84 (3)	2.37 (3)	3.2026 (18)	176 (2)
N2—H2*N*···Cl2	0.94 (2)	2.06 (2)	3.0042 (13)	179.1 (17)
